# Long-Term Nonprogressive Interstitial Lung Disease in Anti-PL-12- and Anti-Ro52-Positive Antisynthetase Syndrome Without Immunosuppressive Therapy

**DOI:** 10.7759/cureus.100677

**Published:** 2026-01-03

**Authors:** Bsher Almaalouli, Harini Reddy Choula, Miguel Rodriguez

**Affiliations:** 1 Internal Medicine, University of Central Florida College of Medicine, Orlando, USA; 2 Rheumatology, SIMEDHealth, Gainesville, USA

**Keywords:** anti-ro52 antibody, antisynthetase syndrome, interstitial lung disease and anti-pl-12 antibody, mechanic’s hands, treatment-free remission

## Abstract

Antisynthetase syndrome (ASS) is an autoimmune condition associated with aminoacyl-tRNA synthetases (ARS) antibodies and often presents with interstitial lung disease (ILD). We describe a patient with ILD and positive anti-PL-12 and anti-Ro52 antibodies. Although these antibodies are generally linked with more active or progressive lung disease, this patient remained clinically stable for several years without immunosuppressive therapy. This case illustrates the broad variability in the clinical expression and course of anti-PL-12-associated disease and shows that some individuals may experience long periods of remission despite serologic profiles often considered high-risk.

## Introduction

Antisynthetase syndrome (ASS) is a rare autoimmune disease marked by antibodies directed against various aminoacyl-tRNA synthetases (ARS), enzymes involved in protein synthesis. Clinically, ASS most commonly presents with interstitial lung disease (ILD), myositis, Raynaud’s phenomenon, joint pain or arthritis, mechanic’s hands, and systemic symptoms such as fever [1]. Among ARS antibodies, anti-Jo-1 is the most prevalent, followed by anti-PL-7 and anti-PL-12 [2-4]. Anti-Jo-1-positive patients typically demonstrate the classic triad of myositis, arthritis, and ILD, whereas anti-PL-12-positive patients more frequently exhibit a lung-predominant phenotype with less frequent muscle and joint involvement [5].

Anti-PL-12-associated ILD is clinically significant due to its higher prevalence, diagnostic complexity, and reported association with more severe or progressive pulmonary disease. Up to 90% of anti-PL-12-positive patients develop ILD, often in the absence of overt myositis, which may delay recognition of an underlying autoimmune etiology. Consequently, approximately 65% of these patients initially present to pulmonologists rather than rheumatologists, increasing the risk of misdiagnosis as idiopathic pulmonary fibrosis and potentially delaying appropriate immunosuppressive therapy [6].

Anti-Ro52 antibodies frequently coexist with anti-synthetase antibodies, including anti-PL-12, and have been associated with an increased prevalence and severity of ILD across inflammatory myopathy-associated ILD cohorts. In several studies, anti-Ro52 positivity has been linked to more aggressive lung disease and worse pulmonary outcomes, further contributing to its designation as a high-risk serologic marker [7].

Although the association between anti-PL-12 antibodies and ILD has been well described in cohort studies, individual case reports detailing long-term nonprogressive disease, particularly in patients with concurrent anti-Ro52 antibodies, remain uncommon in the medical literature. We present a case of a middle-aged woman with anti-PL-12- and anti-Ro52-positive ASS who exhibited isolated ILD with an unexpectedly prolonged period of clinical and radiographic stability over five years without immunosuppressive therapy.

## Case presentation

**Figure 1:**
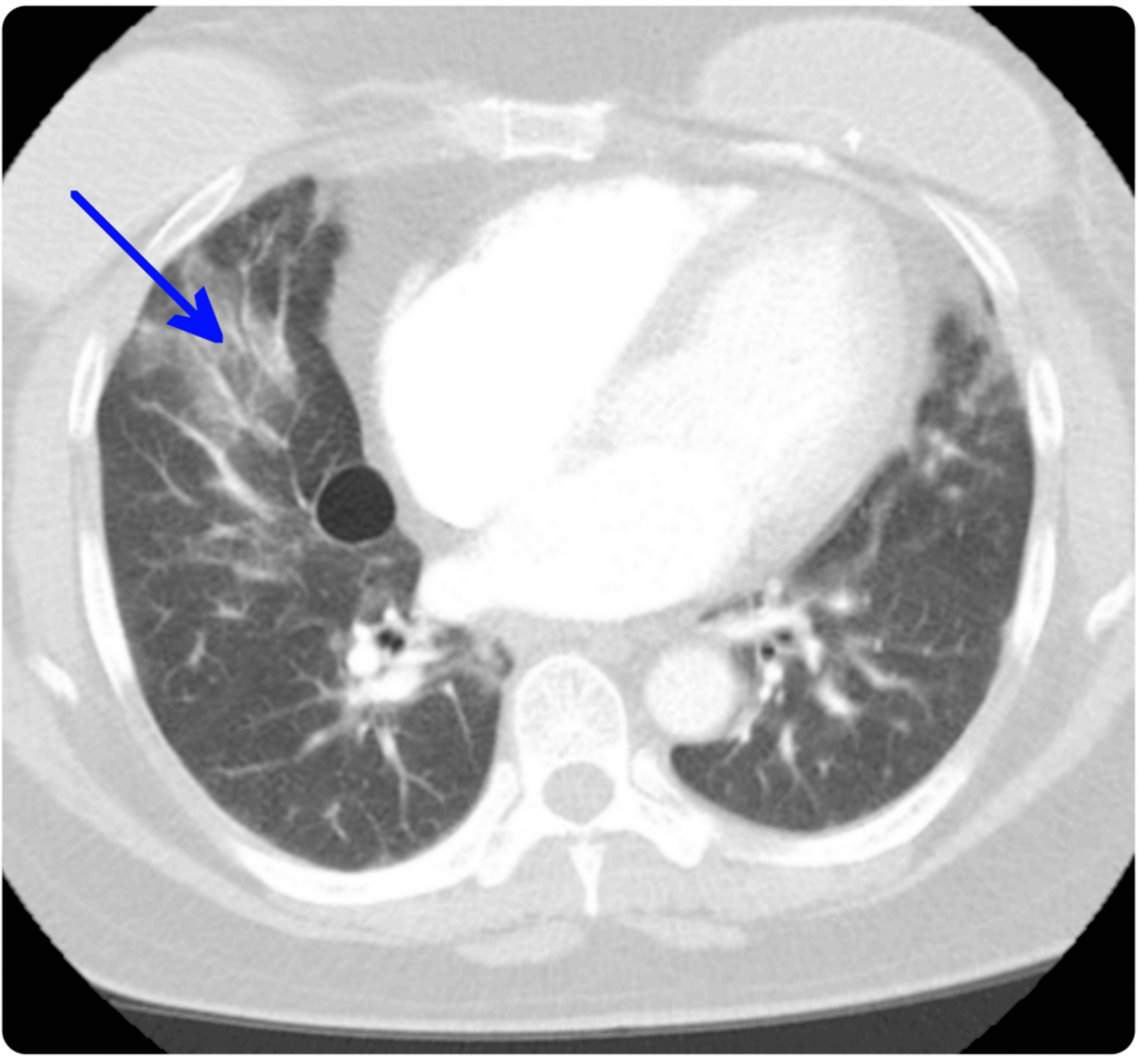
CT image from 2021 showing bilateral ground-glass infiltrates predominantly in the lower lobes, without honeycombing, consistent with active inflammatory interstitial lung disease (arrow).

**Figure 2:**
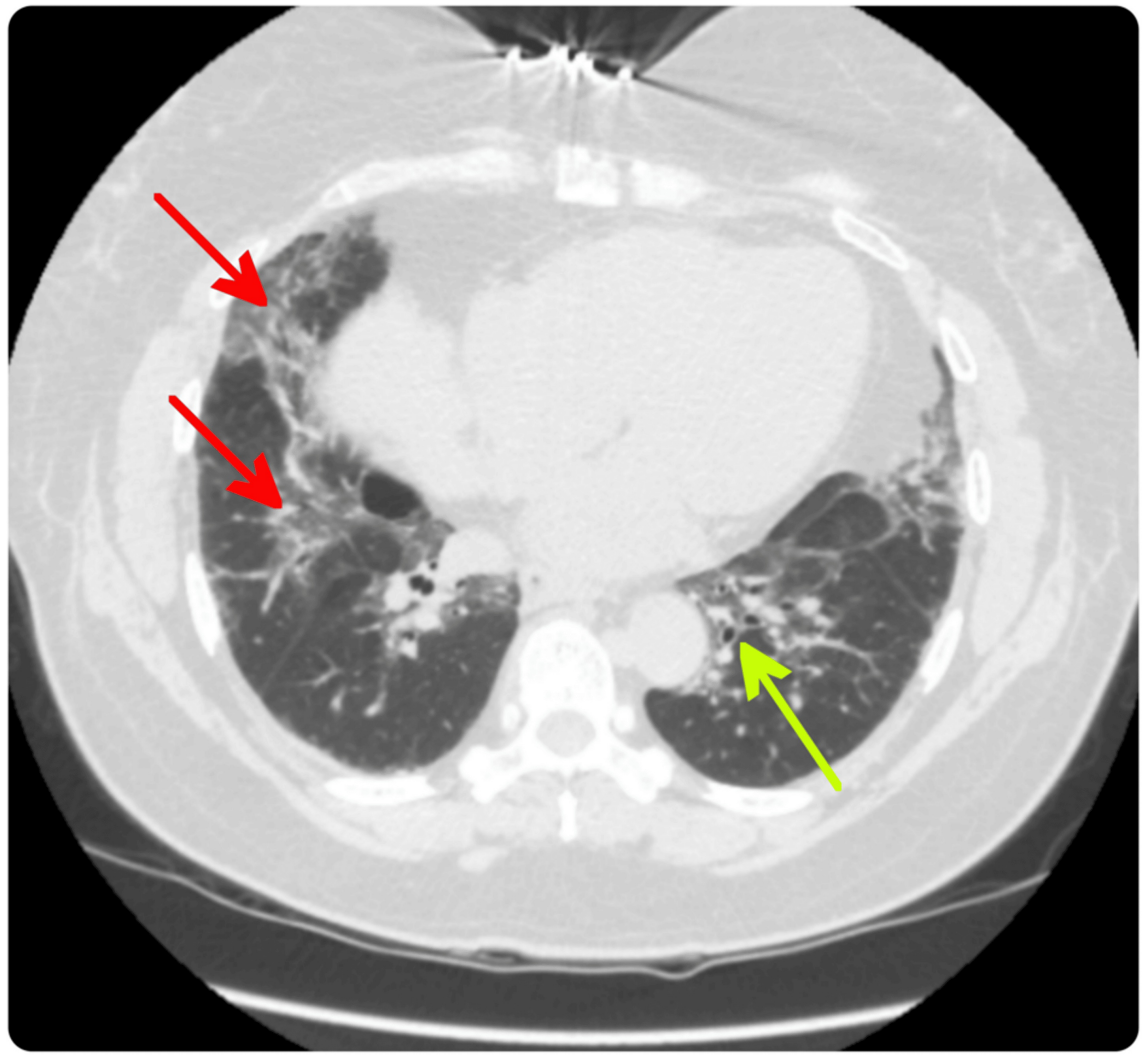
Follow-up HRCT in 2025 demonstrating bibasilar fibrotic scarring (red arrows) and mild bronchiectasis (green arrow), indicating a quiescent phase of connective tissue disease-associated ILD. HRCT: high-resolution computed tomography; ILD: interstitial lung disease

**Figure 3:**
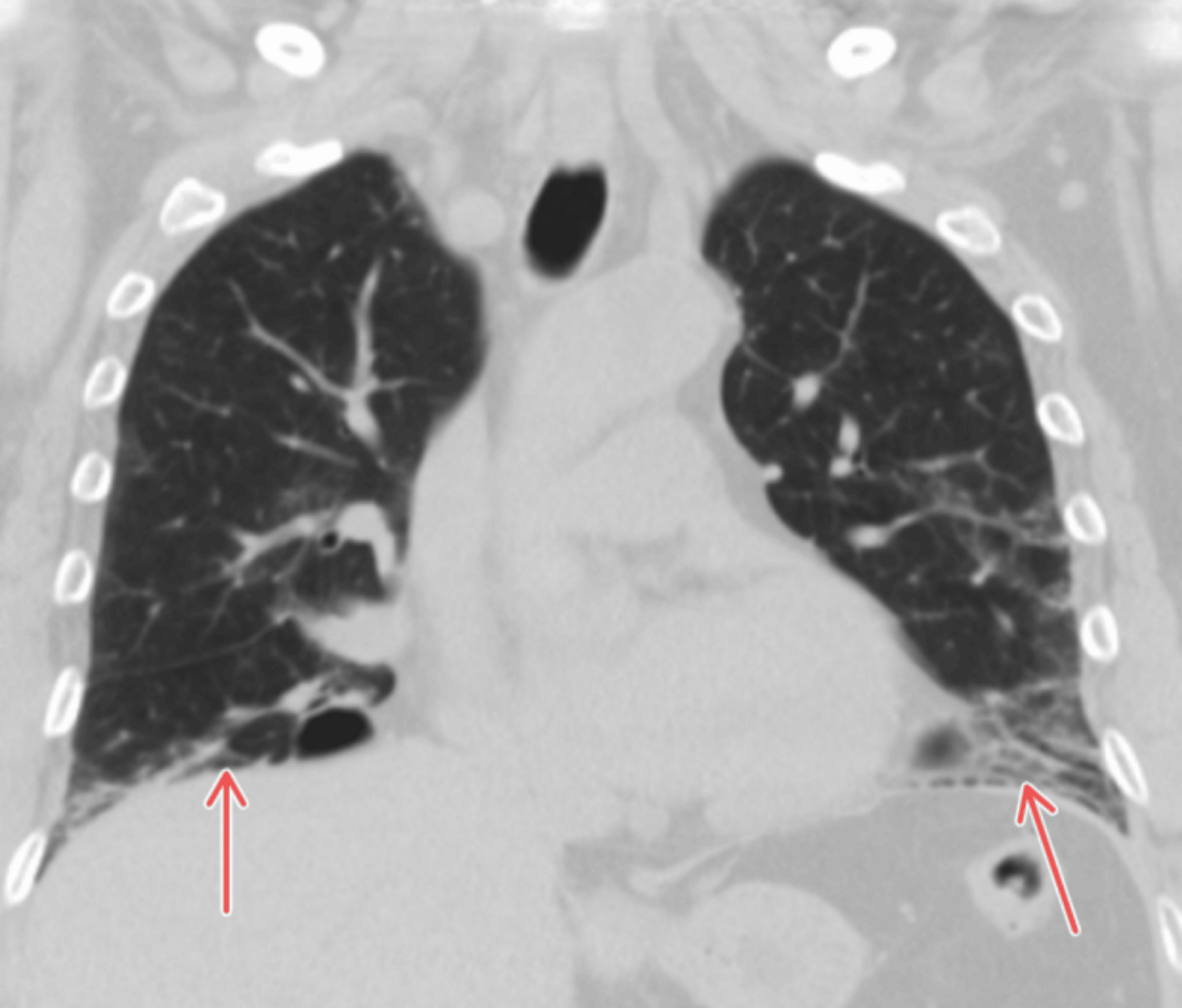
Coronal HRCT in 2025 demonstrating bibasilar fibrotic scarring (red arrows) with reticulation and mild traction bronchiectasis, most consistent with a fibrotic nonspecific interstitial pneumonia pattern in connective tissue disease-associated ILD. HRCT: high-resolution computed tomography; ILD: interstitial lung disease

We report a case of a 56-year-old woman who was referred to the rheumatology clinic following a positive autoimmune workup. She was initially evaluated by a pulmonologist for worsening dyspnea, mainly on exertion, that started four months ago. A high-resolution computed tomography (HRCT) of the chest revealed bibasilar fibrotic changes without significant honeycombing, with resolution of the ground-glass opacities previously seen on imaging in 2021, and no signs of active inflammation (Figures 1-3). Six-minute walk test was normal without desaturation, with oxygen saturation remaining stable at 97%. Pulmonary function testing (PFT) showed a restrictive ventilatory defect and moderately reduced diffusing capacity of the lung for carbon monoxide (DLCO), consistent with underlying ILD.

She had never been diagnosed with COVID-19 or other significant infectious diseases and reported minimal respiratory symptoms for several years prior to presentation, without having received any immunosuppressive therapy. Her autoimmune profile (Table 1) was notable for positive anti-Ro52 and anti-PL-12 antibodies, while other myositis-specific and scleroderma-associated antibodies were negative. On physical examination, she was noted to have mechanic’s hands but no synovitis, rash, Raynaud’s phenomenon, sicca symptoms, or muscle weakness.

**Table 1:** Autoantibody and laboratory results summary ANA: antinuclear antibody; AI: Antibody Index; SI: signal intensity; RNP: ribonucleoprotein antibody; Ro-52: Ro-52 (SSA-52) antibody; U3-snRNP: U3 small nuclear ribonucleoprotein antibody; MDA5: melanoma differentiation-associated gene 5 antibody; OJ: OJ antibody (isoleucyl-tRNA synthetase); EJ: EJ antibody (glycyl-tRNA synthetase); PL-12: PL-12 antibody (alanyl-tRNA synthetase); PL-7: PL-7 antibody (threonyl-tRNA synthetase); PM/Scl-75: polymyositis/scleroderma-75 antibody; PM/Scl-100: polymyositis/scleroderma-100 antibody; Jo-1: Jo-1 antibody (histidyl-tRNA synthetase); Scl-70: scleroderma-70 antibody (topoisomerase I); CRP: C-reactive protein; ESR: erythrocyte sedimentation rate

Test	Result	Reference Range	Unit
Rheumatoid Factor	21	<14	IU/mL
Cyclic Citrullinated Peptide Antibody (IgG)	<16	<16	U/mL
ANA	1:80	Negative or <1:40	Titer
RNP Antibody	1.3	<1.0	AI
RNA Polymerase III Antibody	<20	<20	U/mL
Ro-52 Antibody	136	<11	SI
Th/To Antibody	<11	<11	SI
Fibrillarin (U3-snRNP) Antibody	<11	<11	SI
Centromere B Antibody	<11	<11	SI
Centromere A Antibody	<11	<11	SI
MDA5 Antibody	<11	<11	SI
OJ Antibody	<11	<11	SI
EJ Antibody	<11	<11	SI
PL-12 Antibody	45	<11	SI
PL-7 Antibody	<11	<11	SI
Ku Antibody	<11	<11	SI
PM/Scl-75 Antibody	<11	<11	SI
PM/Scl-100 Antibody	<11	<11	SI
Jo-1 Antibody	<11	<11	SI
Scl-70 Antibody	<11	<11	SI
Mutated Citrullinated Vimentin Antibody	<20	<20	U/mL
CRP	8.1	<5	mg/L
ESR	39	<30	mm/hr
Creatinine Kinase	108	20-300	U/L
Aldolase	3.8	≤7.6	U/L
Immunoglobulin E	5	<100	IU/mL
Absolute Eosinophil Count	152	0-500	cells/µL

The patient underwent repeat PFT four months after the initial rheumatology evaluation, which demonstrated stable spirometry and DLCO values without evidence of physiologic decline. She also had interval follow-up HRCT imaging, which showed no progression of fibrotic changes or new inflammatory features. Clinically, her respiratory symptoms remained stable and well controlled with inhaled bronchodilator and inhaled corticosteroid therapy. Given the absence of progressive symptoms, stable pulmonary function and imaging findings, and lack of extrapulmonary manifestations, the decision to pursue conservative management with close observation was made through a multidisciplinary discussion involving pulmonology and rheumatology. The plan included continued symptomatic treatment, serial PFTs and chest imaging every six months, and close follow-up, with immunosuppressive therapy to be reconsidered if there is evidence of clinical, functional, or radiographic progression.

## Discussion

ASS with positive anti-PL-12 antibodies and ILD typically presents with ILD as the dominant or initial feature, often with minimal or absent myositis and arthritis. Most patients are women, and the median age at onset is in the fifth decade, though younger cases are reported. ILD is present in up to 90% of anti-PL-12-positive patients, and the most common radiographic patterns are fibrotic nonspecific ILD. Anti-PL-12 antibodies account for about 10-14% of ASS cases in large cohorts. Most patients present to pulmonology rather than rheumatology, and isolated ILD is common in this subset. Compared to anti-Jo-1, anti-PL-12-positive patients have less frequent myositis and arthritis, and a higher risk of severe or progressive lung disease. In this case, the patient’s epidemiology and clinical presentation are consistent with the established pattern: a middle-aged woman with ILD as the primary manifestation and only mechanic’s hands as an additional rheumatologic feature. Notably, the patient experienced a prolonged period of clinical stability spanning approximately five years prior to re-evaluation, which is atypical, as most published cohorts report a more progressive or relapsing disease course. This prolonged remission highlights the heterogeneity of disease behavior in ASS-PL12 and suggests that some patients may experience extended periods of stability, even without aggressive immunosuppression [6,8,9].

Multiple studies have shown that anti-Ro52 antibodies are frequently found in patients with ASS, including those with anti-PL-12, and their presence correlates with a higher prevalence and severity of ILD. In idiopathic inflammatory myopathy-associated ILD, anti-Ro52 positivity is an independent risk factor for rapidly progressive ILD and higher mortality. Specifically, patients with both anti-ARS (like anti-PL-12) and anti-Ro52 antibodies have a markedly increased risk of ILD and worse pulmonary function, with some studies reporting up to a 38-fold increased risk of ILD in dual-positive patients compared to those without these antibodies [6,10].

The long-term stability observed in this dual-positive patient highlights an important phenotypic variant within the broader spectrum of ASS. Although anti-Ro52 antibodies are strongly associated with ASS and ILD, their prognostic significance may be less uniform than previously assumed. Pepper et al. demonstrated that, while anti-Ro52-positive patients have a higher frequency of ILD, mortality did not differ between anti-Ro52-positive and anti-Ro52-negative individuals, and ILD severity on imaging and PFT was likewise comparable across antibody groups. These findings suggest that anti-Ro52 identifies susceptibility to ILD but does not necessarily dictate disease trajectory once ILD has developed. The stability in our patient may therefore reflect factors not captured by serologic profiling alone, such as genetic background, younger age at disease onset, immune regulatory differences, or timing of disease recognition, which may mitigate disease progression in certain individuals. Such heterogeneity provides a plausible framework for understanding the unexpectedly stable course seen in our patient despite a high-risk serologic profile [11].

Treatment usually begins with high-dose corticosteroids, often combined with immunosuppressive agents such as mycophenolate mofetil, azathioprine, or tacrolimus. Rituximab is considered for refractory or rapidly progressive ILD [6,12,13]. Early immunosuppression is associated with improved outcomes in severe or progressive cases. However, disease behavior in anti-PL-12-positive ASS-ILD can be heterogeneous, and treatment decisions are generally guided by current clinical, functional, and radiographic activity rather than serologic risk alone. Handa et al. reported delayed fibrosis progression in an anti-PL-12-positive patient who remained stable before eventually requiring immunosuppressive therapy [14]. Similarly, studies describing an indolent ASS-ILD phenotype, including anti-PL-12 cases, suggest that some patients may initially be managed with close observation, although many ultimately require treatment due to later progression [15]. Our patient has remained clinically, physiologically, and radiographically stable for several years without immunosuppression, with minimal symptom burden and no extrapulmonary manifestations. Given the absence of disease progression and the potential risks associated with immunosuppressive therapy, a conservative management strategy with close multidisciplinary monitoring was favored. She continues to undergo serial PFTs and chest imaging, with prompt initiation of immunosuppression should evidence of clinical, functional, or radiographic progression arise.

## Conclusions

This case demonstrates an atypical course of ASS characterized by anti-PL-12 and anti-Ro52 positivity with isolated ILD that remained clinically, physiologically, and radiographically stable for several years without immunosuppressive therapy. This case also highlights the heterogeneity of anti-PL-12-associated ILD and suggests that, in select patients with sustained stability, conservative management with close monitoring may be appropriate. This report underscores the importance of individualized clinical decision-making rather than relying solely on serologic risk profiles.
